# Local excision versus thrombectomy in thrombosed external hemorrhoids: a multicenter, prospective, observational study

**DOI:** 10.1186/s12893-023-02105-4

**Published:** 2023-08-10

**Authors:** Ali Yalcinkaya, Ahmet Yalcinkaya, Semra Demirli Atici, Can Sahin, Sezai Leventoglu, on behalf of T E H Study Collaboration, Alp Yildiz, Hakan Demir, Elif Eren, Furkan Ugur Dundar, Gulsum Sueda Kayacan, Melisa Erdem, Zeynep Nida Ates, Osman Baran Tortum, Zafer Akman, Ahmet Rencuzogullari, Burak Yavuz, Ismail Cem Eray, Ozan Can Tatar, Omer Demirkiran, Sertac Ata Guler, Bulent Calik, Dinc Yaman, Oguz Hancerliogullari, Merve Sakca, Busenur Kirimtay

**Affiliations:** 1 Department of General Surgery, Faculty of Medicine, Gazi University, Ankara, Turkey; 2 Department of Medical Biochemistry, Faculty of Medicine, Hacettepe University, Sıhhiye, Ankara, Turkey; 3 Department of Medical Biochemistry and Microbiology, Science for Life Laboratory, Uppsala University, Uppsala, Sweden; 4 Department of General Surgery, Acibadem Izmir Kent Hospital, Izmir, Turkey; 5 Department of General Surgery, Ankara Yenimahalle Training and Research Hospital, Ankara, Turkey

**Keywords:** Thrombectomy, Local excision, Quality of life, Fecal incontinence, Outcome assessment

## Abstract

**Background:**

Available guidelines describing the procedural treatment of thrombosed external hemorrhoids (TEH) rely solely on expert opinion. We aimed to compare local excision (LE) and thrombectomy (incision) in terms of treatment success, factors affecting success, and outcomes.

**Methods:**

This was a multicenter, prospective, observational study conducted in eight centers from September 2020 to September 2021. A total of 96 patients (58 LE, 38 thrombectomy) were included. Risk factors, demographics and clinical characteristics were recorded. Follow-up studies were scheduled for the 1^st^ week, 1^st^, 3^rd^ and 6^th^ months. Surgical success was assessed at 1 month. Hemorrhoidal Disease Symptom Score (HDSS) and Short Health Scale (SHS) were applied at baseline and the 6^th^ month. Wexner fecal incontinence score was applied at all follow-up studies.

**Results:**

Overall mean age was 41.5 ± 12.7 years. At baseline, groups were similar with regard to demographics and disease severity (HDSS) (p > 0.05 for all). Success was relatively higher in the thrombectomy group (86.8%) compared to the LE group (67.2%) (*p* = 0.054). Constipation and travel history were significantly associated with lower likelihood of LE success. Symptoms during follow-up were similarly distributed in the groups. Both methods yielded significant improvements in HDSS, SHS and Wexner scores; however, SHS scores (6 months) and Wexner scores (all time points) were significantly better in the thrombectomy group.

**Conclusion:**

The in-office thrombectomy procedure may have better short-term outcomes compared to LE in terms of relative success, recurrence and quality of life–despite the fact that success rates were statistically similar with the two interventions. LE may yield particularly worse results in patients with constipation and travel history; thus, thrombectomy appears to be especially advantageous in these patient subsets.

## Introduction

External hemorrhoids, characterized by the enlargement of veins surrounding the anus, are among the most commonly encountered anorectal conditions. They are estimated to have a prevalence of 10–20% with a sharp increase in the 5^th^ decade of life [1–3]. Various risk factors have been identified, including race, family history, pregnancy, lifestyle, working in a sitting position, chronic diarrhea or constipation, rectal surgery, anoreceptive intercourse, and inflammatory bowel disease [3, 4].

Acute thrombosis of the involved vessels is the main complication of external hemorrhoids. Thrombosed external hemorrhoid (TEH) development is usually associated with symptoms of swelling and severe, persistent pain which may be incapacitating for the first several days. Initial treatment is often conservative and consists of bed rest, increased fiber and fluid intake, warm baths (sitz bath), stool softeners, calcium dobesilate, Micronized Purified Flavonoid Fraction (MPFF), analgesics and topical anesthetics [5, 6]. If symptoms are severe or persistent, surgical excision may be offered to patients admitted within 72 h of symptom onset [7]. Today, surgical treatment options for TEH are various, but local excision (LE) and thrombectomy (incision) are recommended approaches [8]. Success is associated with various factors, and postoperative complications and their frequency vary greatly [9]. Currently, there are no definitive guidelines describing the surgical stratification of patients with TEH and available guidelines are based on expert opinion [7, 9, 10], indicating the need for more data concerning the success and complications of surgical treatments in TEH.

The aim of the present study was to evaluate and compare patients who underwent thrombectomy or LE for the treatment of TEH in terms of various characteristics, success, factors associated with success, quality of life, and symptoms and complications during 6-month follow-up.

## Material and methods

### Study design and patient characteristics

This is a multicenter, prospective, observational study conducted with the participation of eight hospitals in Turkey from September 2020 to September 2021. After necessary permissions for the study were obtained, patients admitted with TEH were informed about the study and the study plan was explained. The patient inclusion window lasted for 6 months in each center and all patients were followed up for 6 months after surgical treatment with either LE or thrombectomy. Thrombectomy was performed as an office procedure, while LE was performed in the operating room. Patients were monitored by clinical examinations and/or phone calls scheduled for the 1^st^ week, 1^st^ month, 3^rd^ month and 6^th^ month after surgery.

Individuals who underwent surgical treatment for TEH, accepted to participate in the study and satisfied the inclusion criteria were included in the patient pool. All consecutive patients ≥ 18 years of age undergoing thrombectomy or local excision treatment within the study inclusion window were assessed for enrollment. Patients younger than 18 years of age, individuals with a history of perianal inflammatory bowel disease, Crohn’s disease, ulcerative colitis, diverticular disease, anorectal fistula, colon cancer or other cancers, those with current pregnancy or metabolic disorders, subjects with alcohol or substance addiction, and patients who refused to participate or declined to continue follow-up were excluded from the study. A final total of 96 patients (58 treated with LE, 38 treated with thrombectomy) were included in the study according to inclusion/exclusion criteria.

### Diagnosis, definitions and data collection

After admission to the general surgery departments of the respective hospitals, patients were evaluated clinically for TEH diagnosis. Patients with indications for either LE or thrombectomy, based on the guidelines of the American Society of Colon and Rectal Surgeons and the European Society of ColoProctology, were treated accordingly [7, 10]. As the treatment protocol for TEH is not clearly identified in current guidelines, the choice of treatment for TEH (thrombectomy or LE) was decided by the surgeons and patients. Available information concerning expert opinions were offered to patients and final decisions were based on surgeons’ personal experience and preference, with respect to the condition of the patient. The origin of bleeding was confirmed to be TEH in all cases after meticulous examination for other causes of anal bleeding. Surgical success was defined as remission of symptoms and absence of bleeding or recurrence at 1 month follow-up. Recurrence was defined as sustained re-emergence of some or all TEH-related symptoms or findings at any localization after complete resolution of symptoms with intervention. Patients with symptoms suggesting recurrence were called by phone within 2 weeks of their initial symptom(s), and only those reporting continuation or re-emergence of TEH-related symptoms within this period were identified to have recurrence. Patients with sporadic symptoms were not identified to have recurrence.

Demographic characteristics, comorbidities (diabetes mellitus, hypertension), working in a sitting position (less or more than 4 h daily), body mass index (BMI), and data pertaining to the clinical presentation and features of TEH were recorded, including duration of symptoms, TEH localization, severity of symptoms, presence and type of preoperative and postoperative conservative treatment (high fiber diet, calcium dobesilate, sitz bath, MPFF, topical anesthetic). Specific information on risk factors for TEH was collected, including history of anorectal procedures, skin tags, constipation, straining, benign prostatic hypertrophy (in males), diarrhea, constipation, recent travel history, anoreceptive intercourse, number of pregnancies carried to term (in females), and delivery type (vaginal or cesarean section; in females). The surgery-related characteristics recorded in this study were: surgery duration (minutes), surgery position (prone/jackknife, lithotomy, left lateral decubitus), success (yes, no), and need for prolonged hospitalization after surgery.

Possible postoperative complications including bleeding, pain, infection and anal stricture / stenosis were recorded at each follow-up assessment (1^st^ week and 1^st^, 3^rd^ and 6^th^ month follow-up). The Hemorrhoidal Disease Symptom Score (HDSS) and the Short Health Scale (SHS) was used to evaluate health-related quality of life before surgery and at the 6^th^-month follow-up in all patients. HDSS can range from 0 to 20 points, and higher scores indicate worse disease severity. SHS scores range from 4 to 28 points, and higher scores indicate worse quality of life [11]. To evaluate fecal incontinence, the Wexner incontinence score was calculated in all evaluations during the 6-month follow-up period [12].

### Statistical analysis

The SPSS software (version 25.0) was used for all statistical analyses (IBM, Armonk, NY, USA). A *p* value of ≤ 0.05 was defined as the threshold for statistical significance. The normality of distribution of quantitative variables was tested with the Shapiro–Wilk test. Quantitative variables were described with mean ± standard deviation (SD) values, comparisons between groups were performed with the Student’s *t*-test or the Mann–Whitney *U* test depending on normality of distribution. Paired quantitative variables were compared with the Wilcoxon signed rank test (2-group comparisons) or the Friedman test (> 2-group comparisons). The distributions of categorical variables in study groups were compared via Chi-square tests (continuity correction or Fisher exact test). Logistic regression analyses were performed to identify factors independently associated with surgical success in patients treated with either LE or thrombectomy. The regression models included all parameters that demonstrated *p* values of ≤ 0.100 in univariate analyses comparing successful and unsuccessful patients in the respective groups. Odds ratio (OR) and 95% confidence intervals (95% CI) were calculated for parameters found to be significant in regression analysis.

## Results

Among the 96 patients, 58 had undergone thrombectomy and 38 had undergone LE. Mean age was 41.9 ± 13.8 years in the LE group and 40.8 ± 11.1 years in the thrombectomy group (*p* = 0.733). Overall, 71.9% (*n* = 69) of the patients were males, and male:female distribution was 38:20 in the LE group and 31:7 in the thrombectomy group (*p* = 0.139). Benign prostate hyperplasia was present in 16 of the 69 males, and vaginal birth was reported by 6 of the 27 females. The demographic characteristics, BMI, comorbid diseases, TEH risk factors, HDSS, use of conservative treatment(s) and hospitalizations are summarized and compared according to surgical approach (LE vs. thrombectomy) in Table 1. HDSS results were similar in the two groups (*p* = 0.183). Surgical success with thrombectomy (86.8%) was relatively higher compared to LE (67.2%), but statistical significance was marginally absent (*p* = 0.054). Compared to patients who underwent thrombectomy, those who received LE had significantly higher frequencies of diabetes mellitus (5.3% vs. 24.1%, respectively; *p* = 0.032) and hypertension (7.9% vs. 29.3%, respectively; *p* = 0.023). TEH localization also demonstrated a significant difference between groups; the great majority of patients with LE had multiple (25.9%) or right lateral (51.7%) TEH. Whereas, the most frequent localizations were identified as the posterior (28.9%) and right lateral (21.1%) in the thrombectomy group (*p* = 0.004).

**Table 1:** Patient characteristics according to surgery type

		**Local excision** **(*n* = 58)**	**Thrombectomy** **(*n* = 38)**	***p* value**
Age (years)	(mean ± SD)	41.9 ± 13.8	40.8 ± 11.1	0.733
BMI (weight/height^2^)	(mean ± SD)	25.97 ± 3.86	26.66 ± 3.73	0.205
Surgery duration (min)	(mean ± SD)	28.8 ± 19.7	24.9 ± 14.4	0.622
HDSS	(mean ± SD)	7.12 ± 4.11	5.82 ± 2.91	0.183
Sex	Male	38 (65.5%)	31 (81.6%)	0.139
	Female	20 (34.5%)	7 (18.4%)	
TEH localization	Anterior	3 (5.2%)	5 (13.2%)	**0.004**
	Posterior	6 (10.3%)	11 (28.9%)	
	Right lateral	30 (51.7%)	8 (21.1%)	
	Left lateral	4 (6.9%)	7 (18.4%)	
	Multiple	15 (25.9%)	7 (18.4%)	
Surgery position	Prone/Jackknife	38 (65.5%)	30 (78.9%)	0.087
	Lithotomy	10 (17.2%)	7 (18.4%)	
	Left lateral decubitus	10 (17.2%)	1 (2.6%)	
Surgical success	Yes	39 (67.2%)	33 (86.8%)	0.054
	No	19 (32.8%)	5 (13.2%)	
Working position	< 4 h in a sitting position	16 (27.6%)	12 (31.6%)	0.848
	≥ 4 h in a sitting position	42 (72.4%)	26 (68.4%)	
Diabetes mellitus	Yes	14 (24.1%)	2 (5.3%)	**0.032**
	No	44 (75.9%)	36 (94.7%)	
Hypertension	Yes	17 (29.3%)	3 (7.9%)	**0.023**
	No	41 (70.7%)	35 (92.1%)	
Anorectal surgery history	Yes	4 (6.9%)	1 (2.6%)	0.653
	No	54 (93.1%)	37 (97.4%)	
Family TEH history	Yes	8 (13.8%)	6 (15.8%)	1.000
	No	50 (86.2%)	32 (84.2%)	
Straining during defecation	Never	10 (17.2%)	6 (15.8%)	0.315
	Rarely	18 (31%)	14 (36.8%)	
	Sometimes	18 (31%)	6 (15.8%)	
	Usually	11 (19%)	9 (23.7%)	
	Always	1 (1.7%)	3 (7.9%)	
Constipation	Yes	35 (60.3%)	20 (52.6%)	0.592
	No	23 (39.7%)	18 (47.4%)	
Diarrhea	Yes	4 (6.9%)	1 (2.6%)	0.653
	No	54 (93.1%)	37 (97.4%)	
Anal fissure	Yes	6 (10.3%)	3 (7.9%)	0.964
	No	52 (89.7%)	35 (92.1%)	
Skin tag	Yes	18 (31%)	5 (13.2%)	0.078
	No	40 (69%)	33 (86.8%)	
Travel history	Yes	7 (12.1%)	4 (10.5%)	1.000
	No	51 (87.9%)	34 (89.5%)	
Anoreceptive intercourse	Yes	4 (6.9%)	0 (0%)	0.258
	No	54 (93.1%)	38 (100%)	
Any type of conservative treatment prior to surgery	Yes	38 (65.5%)	24 (63.2%)	0.985
	No	20 (34.5%)	14 (36.8%)	
Hospitalization	Yes	27 (46.6%)	24 (63.2%)	0.166
	No	31 (53.4%)	14 (36.8%)	
Hospitalization duration (days)	(mean ± SD)	9.6 ± 18.6	6.5 ± 9.5	0.381
Any type of conservative treatment after surgery	Yes	51 (87.9%)	29 (76.3%)	0.225
	No	7 (12.1%)	9 (23.7%)	

*p* values in bold indicate the presence of statistical significance

*BMI* body mass index, *HDSS* hemorrhoidal disease symptom score, *TEH* thrombosed external hemorrhoids

Surgery was defined as a success in 67.2% (39/58) and 86.8% (33/38) of patients who had undergone LE and thrombectomy, respectively (*p* = 0.054). BMI was significantly lower in patients with unsuccessful LE (*p* = 0.024). Benign prostate hyperplasia and vaginal birth were found to be unassociated with success in either of the two surgical approaches. Compared to those with successful results, patients with unsuccessful LE had significantly greater frequencies of constipation (48.7% vs. 84.2%, *p* = 0.010) and recent travel history (2.6% vs. 31.6%, *p* = 0.001). In other words, LE was unsuccessful in 16 of the 35 (45.7%) patients with constipation and in 6 of the 7 (85.7%) patients with recent travel history (Table 2). In patients who underwent thrombectomy, the frequency of females was higher in the unsuccessful group (*p* = 0.010), and the prone/jackknife position was associated with greater success compared to the lithotomy and left lateral decubitus positions (*p* = 0.032) (Table 2).

**Table 2:** Comparison of various characteristics and risk factors according to surgical success

**Local excision (*n*** **= 58)**		**Successful (***n* **= 39)**	**Unsuccessful (***n* **= 19)**	***p*** **value**
Age (years)	(mean ± SD)	42.1 ± 13.5	41.6 ± 14.7	0.734
BMI (weight/height^2^)	(mean ± SD)	26.7 ± 3.7	24.5 ± 3.8	**0.024**
Surgery duration (min)	(mean ± SD)	27.5 ± 17.7	31.6 ± 23.7	0.798
HDSS	(mean ± SD)	6.86 ± 3.83	7.73 ± 4.8	0.558
Sex	Male	25 (64.1%)	13 (68.4%)	0.745
	Female	14 (35.9%)	6 (31.6%)	
TEH localization	Anterior	2 (5.1%)	1 (5.3%)	0.748
	Posterior	4 (10.3%)	2 (10.5%)	
	Right lateral	22 (56.4%)	8 (42.1%)	
	Left lateral	3 (7.7%)	1 (5.3%)	
	Multiple	8 (20.5%)	7 (36.8%)	
Surgery position	Prone/Jackknife	29 (74.4%)	9 (47.4%)	0.081
	Lithotomy	6 (15.4%)	4 (21.1%)	
	Left lateral decubitus	4 (10.3%)	6 (31.6%)	
Working position	< 4 h in a sitting position	11 (28.2%)	5 (26.3%)	1.000
	≥ 4 h in a sitting position	28 (71.8%)	14 (73.7%)	
Diabetes mellitus	Yes	8 (20.5%)	6 (31.6%)	0.514
	No	31 (79.5%)	13 (68.4%)	
Hypertension	Yes	11 (28.2%)	6 (31.6%)	1.000
	No	28 (71.8%)	13 (68.4%)	
Anorectal surgery history	Yes	3 (7.7%)	1 (5.3%)	1.000
	No	36 (92.3%)	18 (94.7%)	
Family TEH history	Yes	5 (12.8%)	3 (15.8%)	1.000
	No	34 (87.2%)	16 (84.2%)	
Straining during defecation	Never	8 (20.5%)	2 (10.5%)	0.399
	Rarely	14 (35.9%)	4 (21.1%)	
	Sometimes	10 (25.6%)	8 (42.1%)	
	Usually	6 (15.4%)	5 (26.3%)	
	Always	1 (2.6%)	0 (0%)	
Constipation	Yes	19 (48.7%)	16 (84.2%)	**0.010**
	No	20 (51.3%)	3 (15.8%)	
Diarrhea	Yes	2 (5.1%)	2 (10.5%)	0.446
	No	37 (94.9%)	17 (89.5%)	
Anal fissure	Yes	4 (10.3%)	2 (10.5%)	0.975
	No	35 (89.7%)	17 (89.5%)	
Skin tag	Yes	10 (25.6%)	8 (42.1%)	0.203
	No	29 (74.4%)	11 (57.9%)	
Travel history	Yes	1 (2.6%)	6 (31.6%)	**0.001**
	No	38 (97.4%)	13 (68.4%)	
Anoreceptive intercourse	Yes	2 (5.1%)	2 (10.5%)	0.446
	No	37 (94.9%)	17 (89.5%)	
Any type of conservative treatment prior to surgery	Yes	25 (64.1%)	13 (68.4%)	0.745
	No	14 (35.9%)	6 (31.6%)	
**Thrombectomy (*n* = 38)**		**Successful (*n* = 33)**	**Unsuccessful (*n* = 5)**	***p* value**
Age (years)	(mean ± SD)	41.8 ± 11	34.2 ± 10.2	0.112
BMI, (weight/height^2^)	(mean ± SD)	26.7 ± 3.8	26.2 ± 3.6	0.834
Surgery duration (min)	(mean ± SD)	25.1 ± 15.1	23.8 ± 8.5	0.861
HDSS	(mean ± SD)	6.03 ± 2.87	4.4 ± 3.13	0.557
Sex	Male	29 (87.9%)	2 (40%)	**0.010**
	Female	4 (12.1%)	3 (60%)	
TEH localization	Anterior	5 (15.2%)	0 (0%)	0.752
	Posterior	10 (30.3%)	1 (20%)	
	Right lateral	6 (18.2%)	2 (40%)	
	Left lateral	6 (18.2%)	1 (20%)	
	Multiple	6 (18.2%)	1 (20%)	
Surgery position	Prone/Jackknife	27 (81.8%)	3 (60%)	**0.032**
	Lithotomy	6 (18.2%)	1 (20%)	
	Left lateral decubitus	0 (0%)	1 (20%)	
Working position	< 4 h in a sitting position	10 (30.3%)	2 (40%)	0.664
	≥ 4 h in a sitting position	23 (69.7%)	3 (60%)	
Diabetes mellitus	Yes	2 (6.1%)	0 (0%)	1.000
	No	31 (93.9%)	5 (100%)	
Hypertension	Yes	2 (6.1%)	1 (20%)	0.353
	No	31 (93.9%)	4 (80%)	
Anorectal surgery history	Yes	1 (3%)	0 (0%)	1.000
	No	32 (97%)	5 (100%)	
Family TEH history	Yes	6 (18.2%)	0 (0%)	0.570
	No	27 (81.8%)	5 (100%)	
Straining during defecation	Never	4 (12.1%)	2 (40%)	0.246
	Rarely	14 (42.4%)	0 (0%)	
	Sometimes	5 (15.2%)	1 (20%)	
	Usually	7 (21.2%)	2 (40%)	
	Always	3 (9.1%)	0 (0%)	
Constipation	Yes	18 (54.5%)	2 (40%)	0.653
	No	15 (45.5%)	3 (60%)	
Diarrhea	Yes	1 (3%)	0 (0%)	1.000
	No	32 (97%)	5 (100%)	
Anal fissure	Yes	3 (9.1%)	0 (0%)	1.000
	No	30 (90.9%)	5 (100%)	
Skin tag	Yes	4 (12.1%)	1 (20%)	0.527
	No	29 (87.9%)	4 (80%)	
Travel history	Yes	4 (12.1%)	0 (0%)	1.000
	No	29 (87.9%)	5 (100%)	
Anoreceptive intercourse	Yes	0 (0%)	0 (0%)	N/A
	No	33 (100%)	5 (100%)	
Any type of conservative treatment prior to surgery	Yes	20 (60.6%)	4 (80%)	0.633
	No	13 (39.4%)	1 (20%)	

*p* values in bold indicate the presence of statistical significance

*BMI* body mass index, *HDSS* hemorrhoidal disease symptom score, *TEH* thrombosed external hemorrhoids

Multivariable logistic regression analysis was performed to identify factors independently associated with surgical success. For LE, variables that had a univariate *p* value of ≤ 0.100 were included in the model (BMI, surgery position, constipation and travel history), and the final regression step revealed that presence of constipation (OR: 0.142, 95% CI: 0.028–0.726; *p* = 0.019) and travel history (OR: 0.043, 95% CI: 0.004–0.499; *p* = 0.012) were associated with significantly lower likelihood of success (Nagelkerke R^2^ = 0.353). A similar approach was used for logistic regression in patients who had undergone thrombectomy. The model included sex and surgery position, but neither were found to be independently associated with thrombectomy success.

Next, we evaluated TEH-related symptoms and HDSS, Wexner and SHS scores. Comparisons were performed between study groups (LE vs. thrombectomy) and within study groups (time-bound). Although statistical significance was not present, all patients with recurrence (n = 5) had undergone LE, while none of the subjects in the thrombectomy group experienced recurrence (*p* = 0.153). Both interventions led to a significant decrease in HDSS (*p* < 0.001 for both) and there were no differences between the two groups at baseline (*p* = 0.183) or at 6-months (*p* = 0.138). Both LE and thrombectomy were found to have yielded significant reduction in SHS scores compared to baseline (*p* < 0.001 for both). Baseline SHS scores were similar in the two groups (*p* = 0.054), but postoperative scores were significantly lower in the thrombectomy group compared to the LE group (7.53 ± 4.16 vs. 9.53 ± 4.46, respectively; *p* = 0.006). Finally, Wexner scores demonstrated a significant decreasing trend from the 1^st^ week to the 6^th^ month (*p* < 0.001 for both interventions), and scores were significantly lower at all time points in patients who had undergone thrombectomy (Table 3).

**Table 3:** TEH-related complications, incontinence scores (Wexner) and short health scale scores, within-group and between-group comparisons

		**Overall** **(*n* = 96)**	**Local excision** **(*n* = 58)**	**Thrombectomy** **(*n* = 38)**	***p* value**
Any type of conservative treatment after surgery	Yes	80 (83.3%)	51 (87.9%)	29 (76.3%)	0.225
	No	16 (16.7%)	7 (12.1%)	9 (23.7%)	
Bleeding occurrence within 6 months	Yes	10 (10.4%)	9 (15.5%)	1 (2.6%)	0.083
	No	86 (89.6%)	49 (84.5%)	37 (97.4%)	
Pain occurrence within 6 months	Yes	20 (20.8%)	15 (25.9%)	5 (13.2%)	0.214
	No	76 (79.2%)	43 (74.1%)	33 (86.8%)	
Anal stenosis within 6 months	Yes	2 (2.1%)	2 (3.4%)	0 (0%)	0.517
	No	94 (97.9%)	56 (96.6%)	38 (100%)	
Symptom relapse within 6 months	Yes	6 (6.3%)	4 (6.9%)	2 (5.3%)	1.000
	No	90 (93.8%)	54 (93.1%)	36 (94.7%)	
Infection within 6 months	Yes	1 (1%)	1 (1.7%)	0 (0%)	1.000
	No	95 (99%)	57 (98.3%)	38 (100%)	
Recurrence within 6 months	Yes	5 (5.2%)	5 (8.6%)	0 (0%)	0.153
	No	91 (94.8%)	51 (91.4%)	38 (100%)	
Preoperative HDSS		6.56 ± 3.68	7.12 ± 4.11	5.82 ± 2.91	0.183
Postoperative HDSS (6^th^ month)		1.93 ± 2.62	2.35 ± 3.01	1.37 ± 1.87	0.138
*p* value (repeated measures)		** < 0.001**	** < 0.001**	** < 0.001**	
Preoperative SHS		17.39 ± 5.61	18.28 ± 5.69	16.03 ± 5.28	0.054
Postoperative SHS (6^th^ month)		8.74 ± 4.43	9.53 ± 4.46	7.53 ± 4.16	**0.006**
*p* value (repeated measures)		** < 0.001**	** < 0.001**	** < 0.001**	
Wexner score 1^st^ week		2.26 ± 4.08	3.33 ± 4.84	0.48 ± 0.85	**0.002**
Wexner score 1^st^ month		1.79 ± 3.61	2.76 ± 4.3	0.23 ± 0.76	**0.001**
Wexner score 3^rd^ month		1.58 ± 3.3	2.48 ± 3.91	0.13 ± 0.72	** < 0.001**
Wexner score 6^th^ month		1.33 ± 2.87	2.04 ± 3.39	0.1 ± 0.54	**0.001**
*p* value (repeated measures)		** < 0.001**	** < 0.001**	** < 0.001**	

*p* values in bold indicate the presence of statistical significance

## Discussion

Due to the limitations in current guidelines, the present study was planned in an attempt to assess the value of two interventions, LE and thrombectomy, for the treatment of TEH. The decision for treatment was made according to the most recent clinical practice guidelines [7]. Comparison of patients at baseline showed significant differences between the LE and thrombectomy groups in terms of TEH localization and the frequencies of diabetes mellitus and hypertension, while other characteristics, including demographics, risk factors and disease severity (determined by HDSS), were similar. Evaluation of factors according to surgical success showed that presence of constipation and travel history lowered the likelihood of LE success. Analysis of outcomes showed that, at all follow-up assessments, fecal incontinence (Wexner) scores were lower (better) in patients who had undergone thrombectomy. Similarly, postoperative (6^th^ month) SHS scores were significantly lower (better) in patients who underwent thrombectomy.

The majority of the literature agrees that TEH incidence demonstrates a peak between the 5th and 7th decades of life, with similar frequencies among males and females despite particularly greater prevalence among pregnant women [13–16]. Our data is similar to the literature in terms of age, as demonstrated by ≥ 40 years of age in the study group. However, sex distribution showed a relatively higher proportion of male patients, which is a finding supported by several studies [6, 8]. In addition to surprisingly limited evidence regarding therapeutic success rates, studies comparing the success rates of procedures for TEH are exceedingly rare. This problem becomes particularly evident when the literature is reviewed for reliable studies that have compared therapeutic approaches. In an earlier randomized controlled trial which performed a 1-year follow-up of patients treated by conservative treatment (glyceryl trinitrate), excision, or thrombectomy (incision), the thrombectomy approach was found to result in significantly worse outcomes in terms of pain (short-term, 4 days), symptom remission (1-year), and recurrence (1-year) [17]. Conversely, in the present study, thrombectomy success rate was found to be relatively higher compared to LE, albeit statistical comparison was marginally non-significant (*p* = 0.054). Considering the fact that the aforementioned trial was conducted in 2001, it is possible that the advances in interventional techniques and increased experience may have contributed to the higher success with thrombectomy in our study. The superiority of thrombectomy shown in this study may also stem from the fact that systematic comorbidities (diabetes mellitus and hypertension) were significantly more frequent among subjects in the LE group, thereby causing lower success and greater likelihood of recurrence.

In the present study, the majority of parameters analyzed were similar between the successful and unsuccessful groups in both interventions. However, multivariable analysis revealed that LE success was independently associated with constipation and travel history. The literature concerning factors associated with procedure success in TEH is practically non-existent. Greenspon et al. reported that having a prior history of TEH was the only factor independently associated with recurrence development, but they did not analyze factors associated with treatment success. Other factors included in their model (obesity, constipation, straining, diarrhea, skin tags, anal fissure and internal hemorrhoids) were unassociated with recurrence [6]. To our knowledge, ours is the first study that reports factors associated with success for patients who underwent LE for TEH, and we found that the likelihood of LE success was reduced by sevenfold in patients with constipation (OR: 0.142) and by 23.3-fold in patients with recent travel history (OR: 0.043). When taken together with the relatively higher success of thrombectomy, this result suggests that advising thrombectomy to patients with constipation or travel history could yield better outcomes among patients with TEH.

Evaluation of outcomes throughout the 6-month follow-up period showed that symptoms, frequency of recurrence, and need for conservative treatment after surgery were similar in the two groups. Of note, minor bleeding that did not necessitate intervention was identified in 10 (10.4%) of our patients during follow-up, but this can be explained by the relatively higher tendency of bleeding in recipients of LE (9 of the 10 patients). Despite the marginally non-significant but appreciable difference in surgical success, assessment of recurrence at 6 months showed that 91.4% of LE cases and all (100%) thrombectomy cases were without recurrence. This appears to be in contrast with prior studies suggesting excision to be advantageous in terms of recurrence [17–19]. Our data show that recurrence had developed in 8.6% of patients who underwent LE, which is lower compared to some studies [6], but mostly in agreement with other studies [17, 18]. Based on expert opinion, conventional excision is suggested for the treatment of TEH when applied within 48–72 h of symptom onset [7, 10], and the likelihood of symptom remission and recurrence are reported to be low despite greater postoperative pain [20]. In the present study, the evaluation of scores showed that both treatments resulted in significant improvement in HDSS, Wexner and SHS scores; however, the thrombectomy group had significantly better postoperative Wexner and SHS scores. Relatively worse scores may be associated with the fact that excisional methods can destabilize the anal region and sphincter [21, 22]. These findings indicate that thrombectomy, which is an in-office procedure, may be a better option to reduce the likelihood of incontinence (or related complaints) after treatment, thereby improving postoperative quality of life. In addition, this study showed that being vigilant about the effects of various details may impact the outcomes of treatment, and therefore, careful treatment and close management must be emphasized [23].

This was a multicenter, prospective, observational study including patients from eight hospitals across Turkey, and therefore, we believe the study group accurately represents the general characteristics of the population with TEH. One particular limitation is that the follow-up period was confined to 6 months; thus, the frequency of recurrence may have been underestimated. However, recurrence frequency was in agreement with the majority of the literature, and Jongen et al. reported a recurrence frequency of 6.5% within two months after treatment [18]. Secondly, we excluded pregnant subjects in order to prevent bias regarding factors unique to pregnant females because pregnancy is a well-defined, severe risk factor for TEH. Nonetheless, their exclusion may have caused the relatively lower frequency of females in the study, thereby limiting generalizability to this population. Thirdly, since only subjects who accepted surgical treatment were included, our results may be skewed towards patients with relatively severe TEH who might have been more likely to accept interventions. Additionally, diabetes mellitus and hypertension were both found to be significantly more frequent among subjects who had undergone LE, which may be seen as a cause of biased outcomes regarding success and recurrence. However, the frequencies of these comorbidities were similar among patients with successful and unsuccessful LE, indicating that these comorbidities were unlikely to have had a strong influence on success. Essentially, it is evident that the main limitation of this study was the non-randomization of patients, leading to potential bias in the reported results. As such, our findings should be confirmed by other randomized controlled trials which employ study designs that allow the enrollment of large patient groups [24].

## Conclusion

Our data demonstrate that the in-office thrombectomy procedure may have better short-term outcomes compared to LE in terms of postoperative incontinence, relative success, recurrence and quality of life. These findings and the relatively higher success of the thrombectomy procedure are important to consider when making treatment decisions –despite the fact that success rates were statistically similar with the two interventions. In addition, since travel history and constipation were independently associated with lower likelihood of LE success, suggesting thrombectomy to patients with these characteristics appears to be advisable.

## Data Availability

All relevant data are presented in the article. No further data are available.
