# Correction: Local excision versus thrombectomy in thrombosed external hemorrhoids: a multicenter, prospective, observational study

**DOI:** 10.1186/s12893-023-02215-z

**Published:** 2023-10-24

**Authors:** Ali Yalcinkaya, Ahmet Yalcinkaya, Semra Demirli Atici, Can Sahin, Sezai Leventoglu, on behalf of T E H Study Collaboration

**Affiliations:** 1 Department of General Surgery, Faculty of Medicine, Gazi University, Ankara, Turkey; 2 Department of Medical Biochemistry, Faculty of Medicine, Hacettepe University, S?hhiye, Ankara, Turkey; 3 Department of Medical Biochemistry and Microbiology, Science for Life Laboratory, Uppsala University, Uppsala, Sweden; 4 Department of General Surgery, Acibadem Izmir Kent Hospital, Izmir, Turkey; 5 Department of General Surgery, Ankara Yenimahalle Training and Research Hospital, Ankara, Turkey

**Correction to: BMC Surgery (2023) 23:228**

10.1186/s12893-023-02105-4

In this article [1] TEH Study Collaboration member were incorrectly written tagged and hence was not appearing in Pubmed.

The original article has been corrected.
